# Exploiting Amino Acid Composition for Predicting Protein-Protein Interactions

**DOI:** 10.1371/journal.pone.0007813

**Published:** 2009-11-20

**Authors:** Sushmita Roy, Diego Martinez, Harriett Platero, Terran Lane, Margaret Werner-Washburne

**Affiliations:** 1 Sushmita Roy Computer Science, University of New Mexico, Albuquerque, New Mexico, United States of America; 2 Diego Martinez, University of New Mexico, Albuquerque, New Mexico, United States of America; 3 Harriett Platero Biology, University of New Mexico, Albuquerque, New Mexico, United States of America; 4 Terran Lane Computer Science, University of New Mexico, Albuquerque, New Mexico, United States of America; 5 Margaret Werner-Washburne Biology, University of New Mexico, Albuquerque, New Mexico, United States of America; Cardiff University, United Kingdom

## Abstract

**Background:**

Computational prediction of protein interactions typically use protein domains as classifier features because they capture conserved information of interaction surfaces. However, approaches relying on domains as features cannot be applied to proteins without any domain information. In this paper, we explore the contribution of pure amino acid composition (AAC) for protein interaction prediction. This simple feature, which is based on normalized counts of single or pairs of amino acids, is applicable to proteins from any sequenced organism and can be used to compensate for the lack of domain information.

**Results:**

AAC performed at par with protein interaction prediction based on domains on three yeast protein interaction datasets. Similar behavior was obtained using different classifiers, indicating that our results are a function of features and not of classifiers. In addition to yeast datasets, AAC performed comparably on worm and fly datasets. Prediction of interactions for the entire yeast proteome identified a large number of novel interactions, the majority of which co-localized or participated in the same processes. Our high confidence interaction network included both well-studied and uncharacterized proteins. Proteins with known function were involved in actin assembly and cell budding. Uncharacterized proteins interacted with proteins involved in reproduction and cell budding, thus providing putative biological roles for the uncharacterized proteins.

**Conclusion:**

AAC is a simple, yet powerful feature for predicting protein interactions, and can be used alone or in conjunction with protein domains to predict new and validate existing interactions. More importantly, AAC alone performs at par with existing, but more complex, features indicating the presence of sequence-level information that is predictive of interaction, but which is not necessarily restricted to domains.

## Introduction

Protein interaction networks are networks of physical interactions among proteins and constitute an important component of the bio-molecular network in cells. Capturing the complete set of protein interactions is crucial for understanding the programs for cellular response to different environmental stresses. Although high-throughput technology has advanced our knowledge of proteomes of many organisms [1]–[6], the estimated false negative rates of these datasets suggests a non-trivial fraction of interactions remains undetected [7].

Computational prediction of protein interactions are becoming increasingly popular because they provide an inexpensive way of predicting the most likely set of interactions at the entire proteome scale [8], [9] and can be used to complement experimental approaches. Existing approaches typically use binary classification frameworks that differ in the features used to represent protein pairs. Researchers commonly use static features, such as protein domains [10]–[13], amino acid signatures [14], phylogenetic profiles [15], [16] or, condition-specific dynamic features, such as gene expression [17], or literature-based features such as cellular localization [18].

Protein domains are the most commonly used static features for classification of protein interactions. Although protein domains yield high accuracy classifiers by incorporating evolutionarily-conserved information, these classifiers can only predict interactions between proteins with known domain information. In this paper, we ask the question if we can predict interactions among proteins without relying on domain information, and if so, how complex do our features need to be to perform as well as classifiers using domains as features. In particular, we focus on evaluating classifiers that use simple amino acid composition (AAC) features, which are based purely on normalized counts of single or pairs of amino acids for predicting protein interactions. Approaches that do not rely on domains typically use more complex sequence features comprising 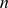-grams (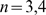) [10], [14], or combine sequence with other sources of information such as gene expression or phylogenetic profiles [19]. While these studies do incorporate amino acid composition for predicting interactions, it is unclear how much predictive power can be obtained from AAC alone.

We performed a systematic analysis of the contribution of AAC to the prediction of protein interactions focussing on different types of datasets (Co-complex, Two-hybrid, Protein Complementation Assay) and classifiers (Maxent, Support vector machines, Naive Bayes). This allowed us to assess the predictive power of AAC over a range of datasets and classifier types.

Interactions predicted in yeast *S. cerevisiae* using AAC were of comparable accuracy to those predicted by protein domains, and other non-domain, but more complex, sequence features. This level of performance suggests that AAC alone can capture a significant amount of information required for interaction prediction. Similar performance was obtained for datasets from higher organisms: fly (*D. melanogaster*) and worm (*C. elegans*). A post-processing analysis of the most important features for interaction prediction identified both domains and AAC features to be important. Some of these AAC features were also statistically over-represented in domains involved in protein interactions.

Finally, we combined predictions from classifiers trained on the three yeast datasets to generate a high confidence yeast interactome. Our predicted interactions had significantly higher tendency to co-express, co-localize, and participate in the same process as compared to the predicted non-interactions, providing expression and gene ontology-based support of our interactions. Our predicted interactions also included several uncharacterized proteins, including a highly connected hub, YJR151W-A, to which we assigned putative functions based on their interaction partners

Overall, AAC has these benefits: (a) AAC is a simple, yet powerful feature which performs surprisingly well given its simplicity, (b) AAC can be used to predict protein interactions irrespective of domain information availability, allowing interaction predictions among uncharacterized proteins for which domain information is scarce, (c) good performance of AAC is independent of the classification framework, and, (d) extraction of AAC features is computationally much more tractable than other non-domain features, making them easily applicable to higher organisms with lengthy protein sequences.

## Results

We first compared AAC against the evolutionarily-rich protein domain features for predicting interactions in the three yeast interaction datasets. We then compared AAC against the tuples and signature product features, which like AAC do not require protein domain information on yeast, worm and fly datasets. We then performed a post-hoc feature analysis to identify the AAC features that were most beneficial for predicting interactions. Finally, we used classifiers combining AAC and domains to predict the complete yeast interactome and validated novel interactions using Gene ontology.

### Comparison with existing features

The goal of comparative analysis was: (a) to determine how well a simple feature like AAC performed against well-known features such as domains, (b) to assess if AAC features can improve performance when used in combination with domains, (c) to compare AAC to other non-domain sequence features such as the tuple feature [10] and the signature product feature [14].

### AAC performs at par with domains

**Figure 1:**
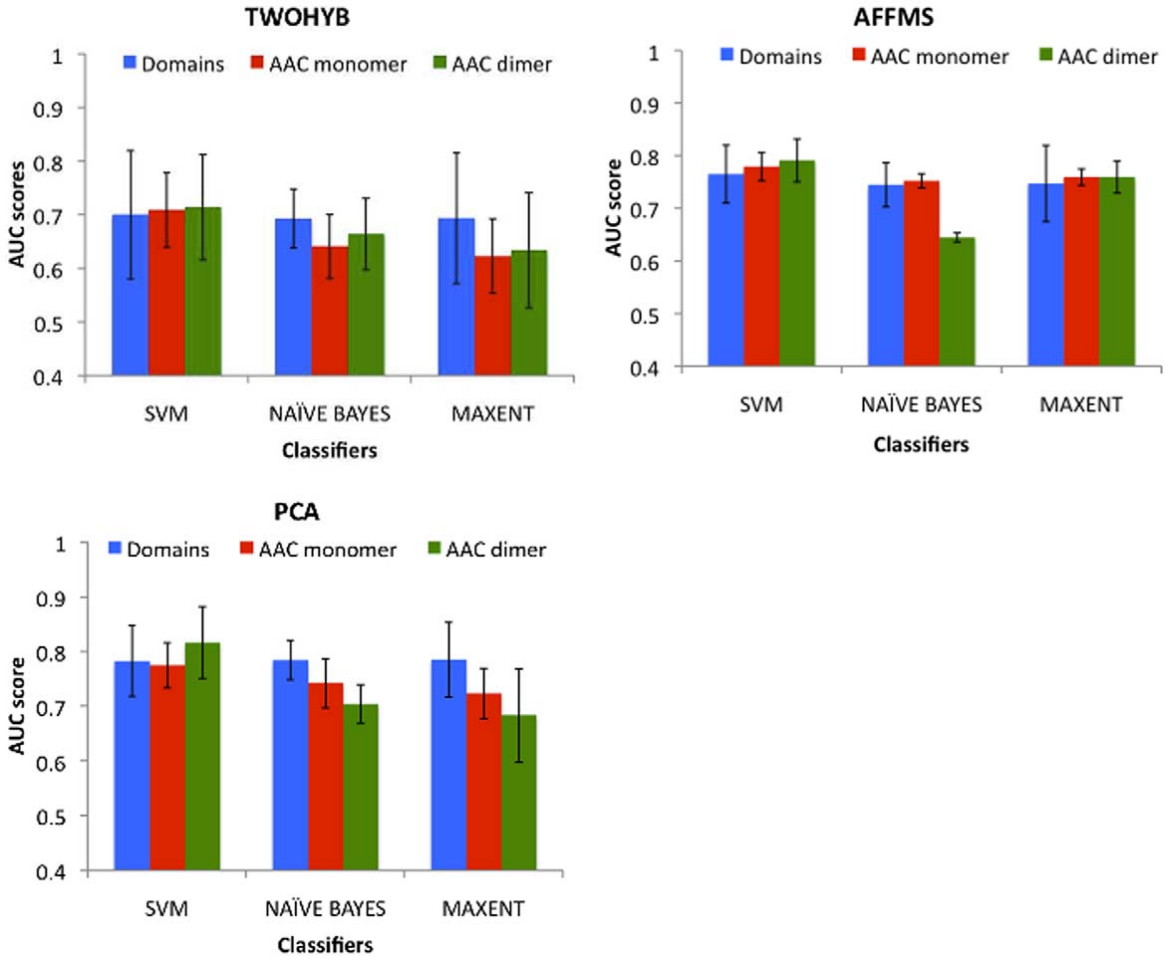
Performance comparison of AAC features (AAC monomer, AAC dimer) against domains. Results are for three classifiers (MAXENT, SVM, NAIVE BAYES) over three yeast datasets (TWOHYB, AFFMS, PCA). The error bars are obtained from five-fold cross validation.

We trained and tested classifiers on the three yeast datasets (TWOHYB, AFFMS, PCA), selecting only protein pairs for which domain information was available for both proteins. We selected only protein pairs with domains to have a fair and direct comparison against a classifier that relies only on domains for interaction prediction (Fig. 1). We compared a classifier using domains against a classifier using either AAC monomers or AAC dimers as features. With the exception of the Naive Bayes using AAC dimer (AFFMS, PCA), surprisingly there was no statistically significant difference in performance of classifiers using AAC features or domains. Overall, both AAC features performed at par with domains in the majority of the cases across different datasets and classifiers, which was surprising and indicated that AAC alone captures a substantial amount of information required for identifying interacting proteins.

### Combining AAC with domains results in no significant improvement in performance

**Figure 2:**
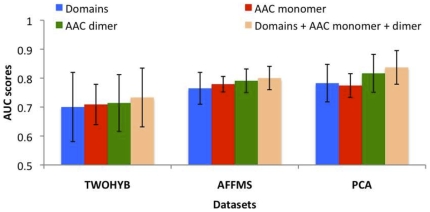
Performance comparison of AAC features in combination with domains. Results are for SVM classifier.

To assess the value of combining AAC with evolutionarily-rich domain features, we combined domains with AAC monomer and AAC dimer features and compared the performance of classifiers using the combined set of features against classifiers using either of these features alone. We estimated performance on the protein pairs for which we had domains to allow comparison against a classifier which used only domains as features (Fig. 2). In all three datasets, combining AAC with domain features did not significantly change performance, which is not surprising because the protein pairs have domains and therefore should be highly predictable using the domain-based classifier. This results suggests that we can safely combine simple sequence-based features with evolutionarily-conserved features such as domains without suffering any performance loss due to excessive features.

### AAC performs at par with non-domain features

We compared the performance of AAC with other non-domain features, which can also predict interactions between proteins lacking domain information. The two features that we evaluated were the tuple feature from Gomez *et al.* [10], and the signature products (Sigprod) from Martin *et al.* [14].

We compared AAC against the tuple and Sigprod features on three yeast datasets (TWOHYB, AFFMS, PCA). The three datasets were each split into two parts: protein pairs with domains and protein pairs without domains. We report the performance on protein pairs with domains (With domains), on protein pairs without domains (No Domains) and on the complete dataset (All Protein pairs). The AUC-scores on protein pairs without domains evaluated how well non-domain features including AAC are able to predict interactions (or non-interactions) among proteins for which no domain information is available. The AUC-scores on the complete datasets evaluated the overall performance of different features on protein pairs irrespective of domain information availability. These results are for the SVM classifier, because it provides performance numbers for all features (Sigprod is specific to a SVM classifier). Results for the Maximum entropy classifier are similar (Supporting text S1, Fig. S4).

**Figure 3:**
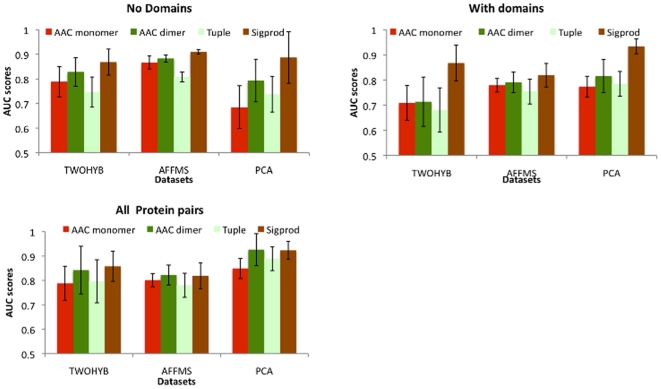
Performance of AAC features against other non-domain features. Non-domain features (Tuple, Sigprod) were compared on protein pairs with domains (With domains), pairs without domains (No domains) and on the entire dataset (All protein pairs).

On protein pairs without domains (Fig. 3), AAC dimers were significantly better (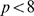
E-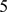) than tuples for AFFMS. AAC monomer was also better than tuples for AFFMS (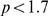
E-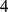). Tuples were never significantly better than the AAC features. AAC features also performed at par with Sigprod with the exception of AAC dimers for AFFMS. This indicates that for these protein pairs, AAC dimers capture the majority of the information captured in Sigprod.

On protein pairs with domains (Fig. 3), Sigprod outperformed AAC features less often (PCA for dimers, PCA, TWOHYB for monomers) than tuples. Both AAC features were much closer to Sigprod than Tuple, especially on the largest dataset (AFFMS). Finally, on the complete datasets, AAC dimers were at par with Sigprod in all three datasets, whereas AAC monomers were at par with Sigprod in two datasets. Overall, AAC features were better than tuples and AAC dimers were at par with the Sigprod features in the majority of the cases.

### Performance comparison on fly and worm datasets

**Figure 4:**
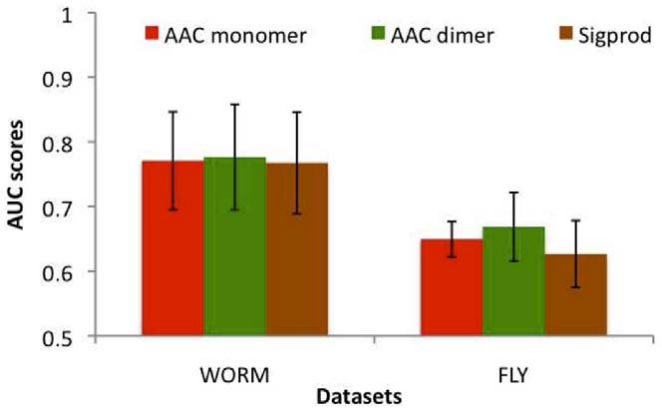
Comparison of AAC features against signature product features (Sigprod) on protein interaction datasets from worm and fly.

In addition to comparing AAC features on the three yeast datasets described above, we also compared AAC features on two-hybrid datasets from worm and fly (Fig. 4). We considered AAC features against the Sigprod features and found that AAC features performed at par with Sigprod. Overall, these results suggest that features based on AAC can perform as well as existing sequence-based features, which do not require domains. This level of performance of AAC features is true for different organisms and different datasets.

### Identification of important features

The fact that AAC monomer and dimer features can have at par performance with more complex features such as tuples, domains or signature product is very surprising considering the simplicity of these features. To investigate what makes AAC a good feature for protein interaction prediction, we considered a classifier using both domains and AAC features and obtained the AAC features that occurred among the 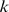 features most important for interaction prediction. We then asked if there were any AAC monomers and dimers that were statistically over-represented in known protein interaction domains [20].

**Figure 5:**
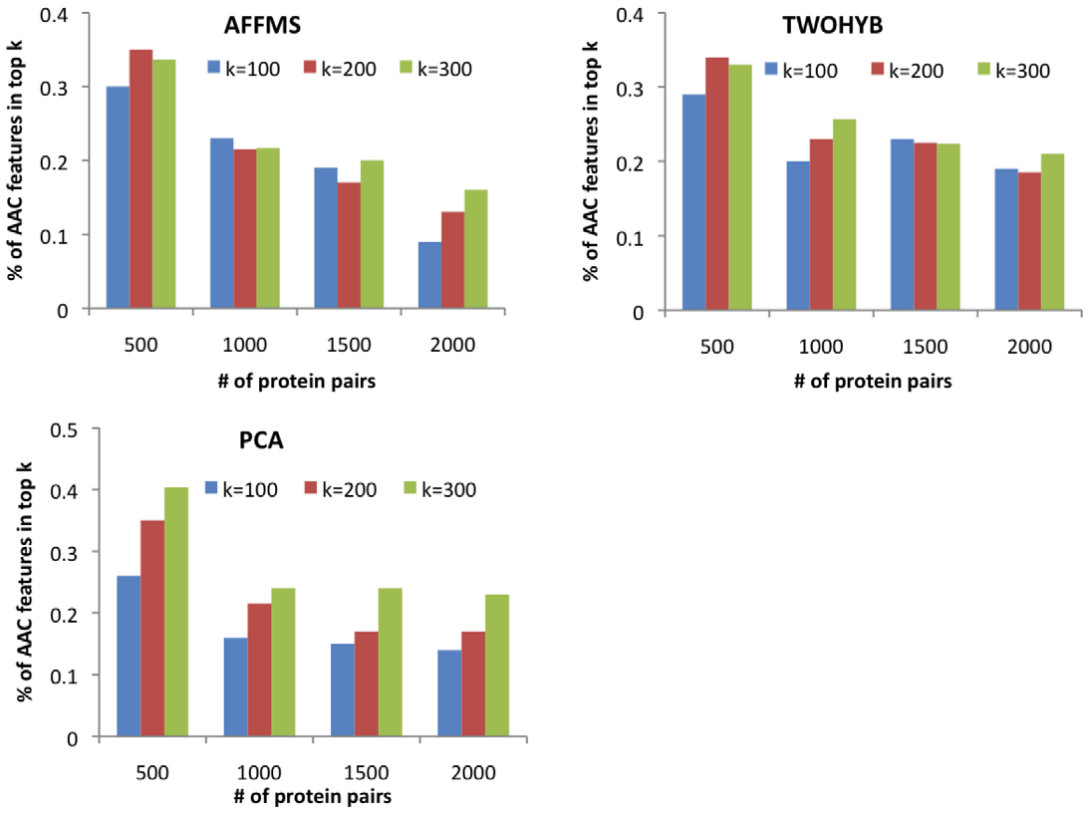
Percentage of the AAC features among the top 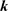 features. The top 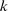 features, 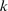 equals 100, 200 or 300, were obtained from correctly predicted protein pairs per dataset (AFFMS, TWOHYB, PCA). The number of correctly predicted proteins pairs were obtained from the most confident 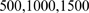 and 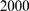 predicted interactions.

We considered the true positives among the top 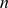, 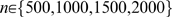 predicted protein pairs and obtained the 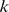, 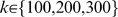 most important features for each set of true positives. These predictions were obtained from classifiers using both AAC and domains features. AAC features comprised 10–40% of the top 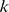 features (Fig. 5). The highest proportion of AAC features were from 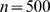, decreasing with larger 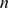, suggesting that AAC contributes to the highest confidence predictions.

**Figure 6:**
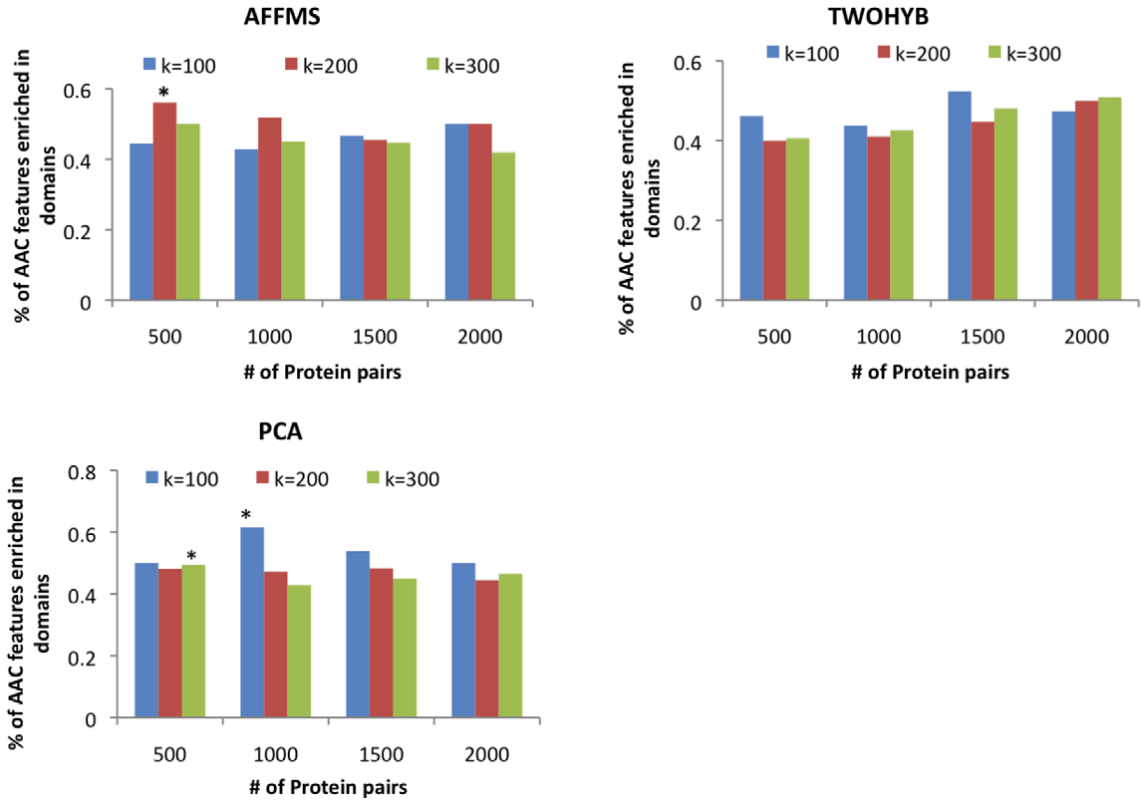
Percentage of the top AAC monomers and dimers that were significantly enriched in domain regions involved in protein interactions. 
 indicates significant overlap (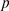-value
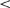
0.05) with the complete set of AAC monomers and dimers found to be over-represented in domains involved in protein interactions.

We found that several of the AAC monomer and dimers were statistically over-represented in regions representing protein-protein interaction domains (Fig. 6). Features differing in discretization levels were considered the same. For example A_1 and A_2 were both considered as Alanine, where 1 and 2 represent discretization level. We assessed statistical significance of observing this proportion of AAC monomers and dimers to be over-represented in the protein interaction domains, by comparing the proportion to the total number of possible AAC monomers and dimers that are enriched in the interaction domains. Of the 420 possible monomers and dimers, there are 175 that are statistically over-represented in protein interaction domains. We found that the proportion of over-represented features was statistically significant for some (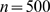, 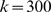, hyper-geometric 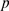-value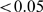) but not all cases. The AAC monomers and dimers that are over-represented are likely capturing crucial information in domains and therefore helping interaction prediction. However, the proportion of AAC monomers and dimers over-represented in domain regions is not always significant, suggesting that overall AAC composition may be capturing additional interaction-sensitive information outside of protein domains.

**Figure 7:**
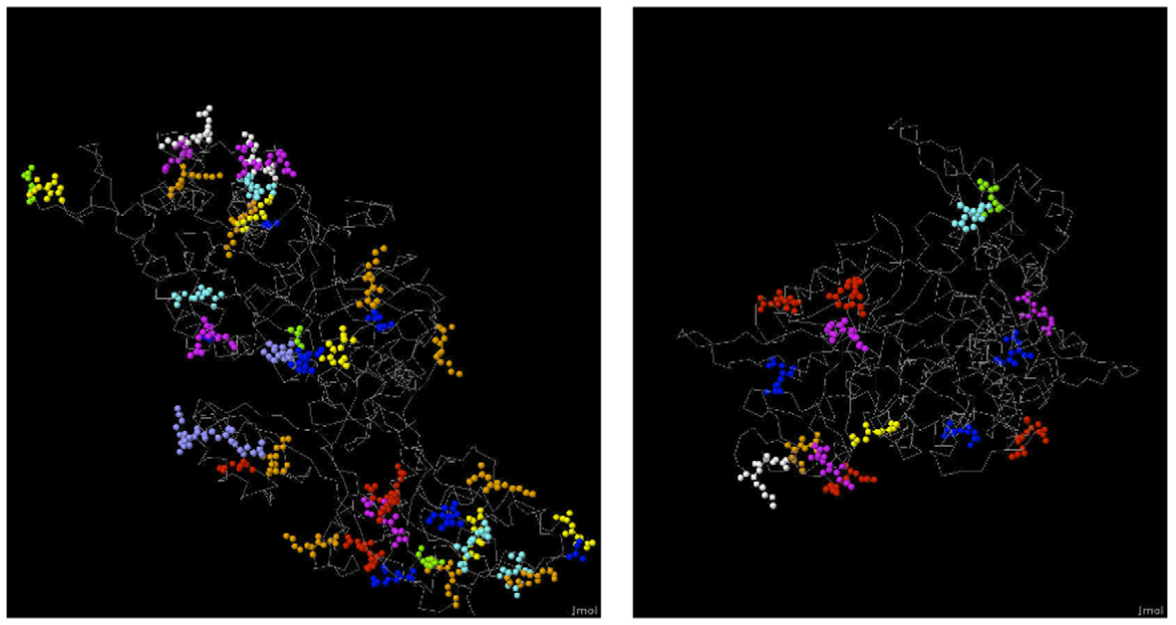
Three dimensional structures of ETF2 and RNR1 proteins obtained from the protein data bank. Only dimers important for prediction are shown with the rest of the protein structure as *backbone*. Different colors correspond to different dimers. EE: red, AE: green, AD:blue, DA: yellow, DE: magenta, DV: cyan, EK: white, EQ: violet, KA: orange.

To visually illustrate that the dimers were capturing meaningful information of interactions we considered two proteins, EFT2 from the high-confidence interacting pairs, and RNR1, from the high-confidence non-interacting pairs (Fig. 7). We selected these proteins from the AFFMS dataset, such that the proteins had roughly the same length and had an associated structure in the protein data-bank covering 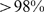 of the protein. We then displayed a select set of dimers that had high scores. We found that several of the dimers differed in concentration (KA, EQ) between the two proteins indicating that the dimers were capturing information discriminating between interacting and non-interacting proteins. Although this is one specific case of all the proteins in interactions or non-interactions, we found this visual differentiation between the different protein types to be encouraging and opens up directions of future research relating 3D structure of proteins and dimers concentrations.

**Figure 8:**
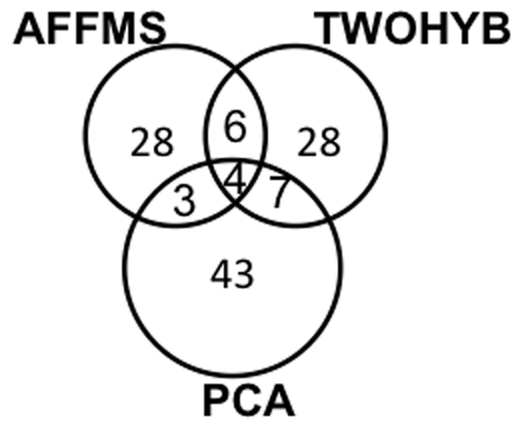
Overlap of AAC monomers and dimers from different datasets.

We examined the overlap between the statistically over-represented AAC monomers and dimers from the three datasets using 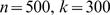. We selected 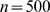 because the percentage of AAC features among the top 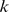 features was maximal for 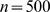. We selected 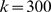 to include a large number of features for comparison. There was not much overlap suggesting that these datasets were capturing different sets of protein interactions (Fig. 8, Table 1). The small overlap set included both hydrophilic (Tyrosine (Y), Tryptophan (W)) and hydrophobic amino acids (Alanine (A), Isoleucine (I)). We found slightly more overlap in TWOHYB and PCA than either with AFFMS, which is not surprising because both PCA and TWOHYB are pairwise interaction sets whereas AFFMS is a co-complex interaction set. Features common to AFFMS and TWOHYB included charged amino acids (Aspartic acid (D), Glutamic acid (E)) and mostly non-polar amino acids (Valine (V), Phenylalanine (F), Alanine (A)). The features common to PCA and AFFMS included charged (Arginine), but mostly non-polar amino acids (Glycine (G), Leucine (L), Tryptophan (W)). Finally, PCA and TWOHYB had features with mostly non-polar amino acids with the exception of one non-polar (Tyrosine, (T)). The identification of primarily non-polar amino acids is somewhat surprising, since it is the polar amino acids that are on the surface of proteins and thought to participate in protein interactions. Thus, the features identified here must be related to some other characteristic of the interacting proteins.

**Table 1:** AAC monomers and dimers over-represented in protein interaction domains.

Dataset combination	AAC Features
AFFMS, TWOHYB, PCA	A I W **Y**
AFFMS, TWOHYB	V A**D** **D**A **E**V F**E** **Q**A
AFFMS, PCA	L G**R** WG
TWOHYB, PCA	FL G GL LA LG WA W**T**
AFFMS	A**E** AM A**R** **D**V **E**G GG G**H** I**Q** **K**A **K**V **R**A**R**V V**E** V**K** V**Q**
TWOHYB	FG FI FV GM I**D** IF IV MA MG **Q**I VG
PCA	AA AF AI AV CF CM F FA FF F**Y** G**D** GF GW IG LF LL LM L**Y**
	**Q**V **RD** **T**G **T**V VC VF V**R** WC WM W**S** **YR**

AAC features enriched in domains in different combinations of the three datasets. Each row represents the features that were exclusive to the dataset combination in the first column. A: Alanine, C: Cysteine, D: Aspartic acid, E: Glutamic acid, F: Phenylalanine, G: Glycine, H: Histidine, I: Isoleucine, K: Lysine, M: Methionine, Q: Glutamine, R: Arginine, T: Threonine, V: Valine, W: Tryptophan, S: Serine, Y: Tyrosine. Bold indicates polar, and underline indicates charged. Non-bold indicates non-polar.

Features that were exclusive to AFFMS included all the charged amino acids (D, E, K, R, H), and one polar (Q) and remaining non-polar amino acids (A, G, M, V, I). In contrast PCA and TWOHYB had very few charged amino acids, only Aspartic acid (D) in TWOHYB, and Aspartic acid (D) and Argnine (R) in PCA. The presence of all charged amino acids in the AFFMS suggests charge may be important for forming large protein complexes. Features exclusive to TWOHYB, had only one polar (Glutamine, Q) amino acid and the remaining were all non-polar (F, G, I, V, M). Finally, features exclusive to PCA included polar (Q, T, Y), charged (D,R) and non-polar amino acids (A, F, C, M, V, W). Overall PCA had the maximum range of amino acids, even though it was the smallest data set.

Our post-hoc analysis of important features led us to conclude that several AAC monomers and dimers were significantly enriched in domains involved in protein interactions, but the specific features that were deemed important depended on the dataset: features involving charged amino acids in AFFMS, and non-polar amino acids in TWOHYB and a mixture of polar and non-polar amino acids in PCA.

### Whole yeast proteome analysis: Identification of novel interactions

To predict interactions in the entire yeast genome, we trained three classifiers on the AFFMS, PCA and TWOHYB datasets. The predicted interactome was created from the intersection of the interaction sets predicted by each classifier. We considered intersections at different confidence levels, ranging in 80%–95%, and identified the number of known interactions at each confidence level (Table 2). We found a large proportion of our interaction set to comprise novel interactions.

**Table 2:** Number of predicted and true interactions at different confidence levels.

Confidence level	Predicted	Known	Predicted + self loops	Known + self loops
0.95	1144	86	1412	197
0.90	4352	194	4862	373
0.85	9769	313	10495	532
0.80	17084	449	18030	708

Number of predicted (using ACC and domains) and known interactions, where known interactions are those present in either AFFMS, TWOHYB or PCA.

**Figure 9:**
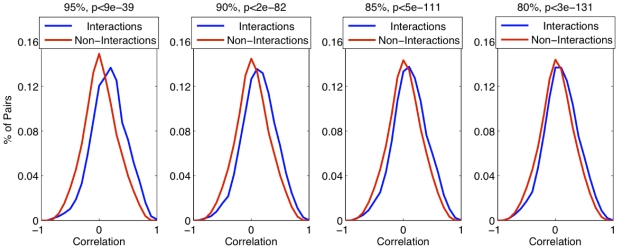
Distribution of co-expression of predicted interactions and non-interactions at different confidence levels. Co-expression is measured by Pearson's correlation coefficient.

Because many of our interactions were novel, we carried out preliminary validation using expression data and gene ontology categories [21]. Our expectations were that interacting proteins would tend to be co-expressed and be in similar processes or locations. For co-expression analysis we computed the correlation coefficient between the two proteins of a predicted interaction (or non-interaction) using expression data from Gasch *et al.* [22], which profiled the transcriptomic response of yeast cells under different stress conditions (Fig. 9). We found that the average correlation for the interactions (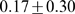), while low, is higher than the non-interacting proteins (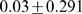, Kolmogorov Smirnov 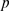-value
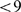
E-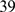). This low correlation has been seen before and suggests that protein stability, maintained via post-translational modifications, may play a significant role in complex formation and function [5], [23], or may be due to proteins interacting under conditions not captured in the expression dataset. However, compared to non-interacting proteins, the interacting proteins exhibit a significant bias in the distribution towards positive correlation.

**Figure 10:**
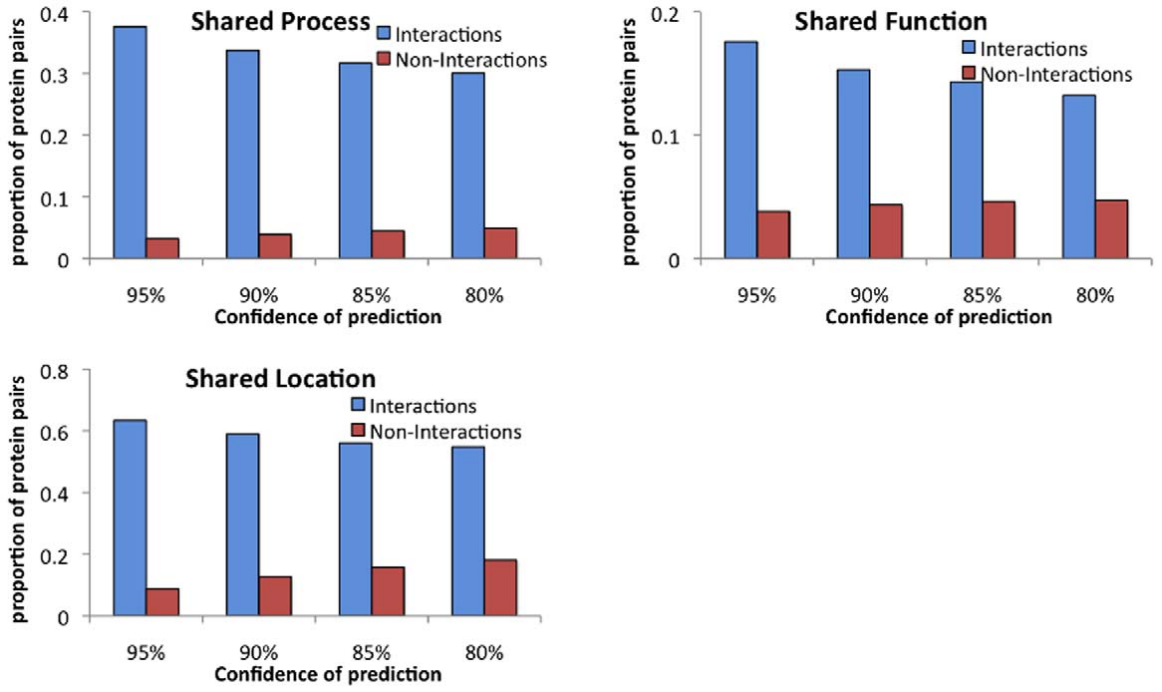
Co-annotation of predicted interactions and non-interactions at different confidence levels of interaction. Predicted interactions and non-interactions at different confidence levels were analyzed for different types of co-annotation: co-process, co-location and co-function.

We further analyzed these interactions for co-localization, co-function, and co-process using GO Slim terms and found that proteins predicted to interact tended to co-localize, or participate in the same processes more than the proteins predicted to not interact (Fig. 10). In particular, interacting proteins were statistically enriched for co-localization (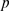-value
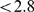
E-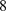) and co-process (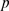-value
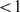
E-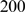) where as predicted non-interactions were statistically depleted from co-localization (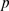-value
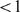
E-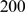) and co-process (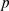-value
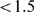
E-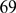). For function, even though predicted interactions had a higher fraction of interactions participating in the same function, both interacting and non-interacting proteins were enriched for co-function. This suggests that GO slim functional categories may not be as predictive of interacting versus non-interacting proteins as process and location. This is consistent with low sensitivity of protein interaction identification using all GO molecular functions versus sensitivity using a filtered set of functions [24]. To investigate this further we considered the enrichment on a per functional category basis and found that both interacting and non-interacting proteins were enriched in hydrolase activity, and non-interacting proteins were enriched in transferase activity. Further, on excluding these two categories, the non-interacting proteins were no longer enriched in co-function while the interacting proteins remained enriched in co-function (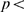
1.7E-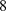). This suggests that proteins that are hydrolases may be further grouped into other categories, some of which interact and some of which do not interact. Proteins that are transferases do not interact with each other. This gives us an interesting direction of future research to investigate the propensity of different proteins to interact based on their functional roles. The high enrichment of co-localization and co-process is consistent with our prediction of interactions and, validates our predicted interactions using gene ontology, and future experimental validation of the high confidence predictions are likely to yield true positive interactions.

### Analysis of novel interactions: Identification of new function

We identified 1412 high confidence (95%) interactions, including 197 existing interactions. We examined more closely the most highly connected nodes (hub nodes) of this high confidence network, where a hub was a node with 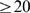 interaction partners. The largest hub was the protein LAS17, an actin assembly protein and the yeast homolog for the Wiskott-Aldrich disease in humans [25]. This protein has 12 known interactions in the existing interaction databases and we found 176 more interactions, most of which were among proteins involved in actin cytoskeleton organization, consistent with the known function of LAS17.

Gene ontology enrichment of the hubs identified cell budding, cytokinesis and mRNA stability and catabolism as additional enriched processes. Other protein hubs were also involved in a variety of processes including nuclear transport (KAP95, SRP1), transcription (NOT3, NAB3) and telomere maintenance (GAL11, STO1). Because hubs captured the majority of the interactions, we concluded that interactions in the high confidence network were involved in cell-budding, actin assembly, nuclear pore transport and mRNA stability.

**Figure 11:**
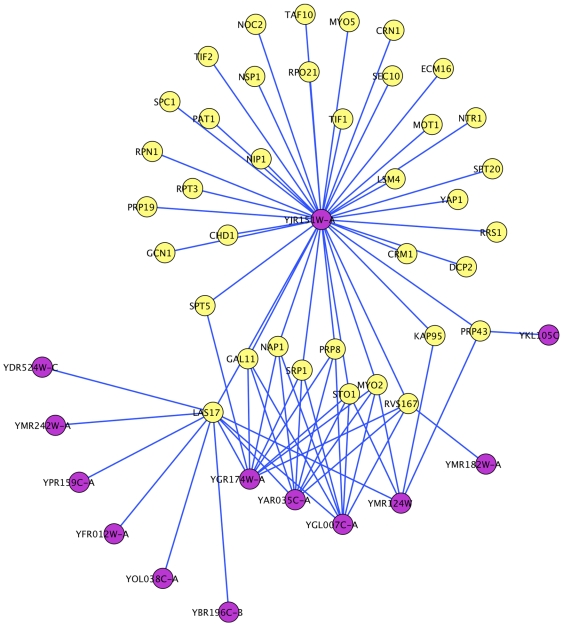
Protein interaction sub network with the uncharacterized ORFs. The uncharacterized ORFs are in magenta and the characterized are in yellow.

One of our goals, using sequence-based interaction classifiers, was to capture and analyze interactions among proteins that cannot be analyzed using domain-based methods. This is especially useful for *uncharacterized* proteins for which roles may be inferred based on interacting proteins. Therefore we focused on predicted protein pairs where one of the proteins did not have any known domains. There were a total of 169 such interactions including 75 interactions involving 13 uncharacterized proteins. One of the uncharacterized proteins (YJR151W-A) was also a hub with 37 interaction partners (Fig. 11). Using the “guilt by association” approach we predict that this protein has a role in transcription, because of its predicted interactions with several universal transcription initiation factors (TIF and TAF), and also in mRNA processing and metabolism, because of its interactions with splicing factors, P-body, and translation-initiation proteins [26]. Interestingly, YJR151W-A may not have been studied carefully because it was not thought to be a gene. We assigned putative roles to other uncharacterized proteins based on their interactions with other characterized proteins (Table 3). The ability to assign new putative function to uncharacterized proteins, for which domains are also not available, highlights the usefulness of predicting protein interactions using non-domain features such as AAC. Overall our interaction set had both known and uncharacterized proteins, allowing us to validate existing knowledge and predict new function for uncharacterized proteins.

**Table 3:** Predicted function of uncharacterized ORFs.

ORF	Degree	Putative function
YJR151W-A	37	protein-RNA complex assembly, mRNA processing, asexual reproduction
YGR174W-A	9	cell budding, asexual reproduction
YAR035C-A	8	cell budding, asexual reproduction
YGL007C-A	8	cell budding, asexual reproduction
YMR124W	5	organelle organization and biogenesis

Degree specifies the number of interaction partners of a protein.

## Discussion

We have described a novel sequence-based feature, amino acid composition (AAC), that can be used to predict protein interactions in different organisms. Compared to other sequence-based features, AAC is much simpler because it models very little sequential dependencies (domains and tuples) and no explicit pairwise information (Sigprod). Surprisingly, despite its simplicity, AAC performs at par with domains on protein pairs for which domain information is available. The good performance of AAC, in spite of its strong independence assumptions, maybe due to its similarity to the *bag of words* model, which often performs at least as well as models that do not make independence assumptions [27].

Compared to tuple features, AAC gave better performance, which was surprising because tuples incorporate ordering information of sequential amino acids. A possible explanation is that grouping of amino acids into six categories, may be too coarse, and by doing so, the tuple features are excluding information specific to individual amino acids, crucial for characterizing protein interactions. Comparison to Sigprod indicated that AAC performed at par on protein pairs without domains, and also on the complete set of protein pairs including those without domains.

On protein pairs with domains, Sigprod features are the best, outperforming all other features (including domains) on at least one dataset. The fact that Sigprod outperforms AAC features is not surprising because it captures more sequential dependency by looking at trimers rather than dimers or monomers. A natural extension of the AAC features would be to look at trimers. However, Sigprod outperforms even domains, which is very surprising because domains represent much longer portions of the amino acid sequence. It is possible that protein interactions do not involve the complete domain, but specific contact points within the domains. Dimers and trimers (Sigprod) are able to capture these crucial contact point information thus providing good performance. Sigprod also uses a specialized string kernel, which gives it additional benefits and therefore improved performance. In contrast, we use AAC features with the general purpose Gaussian kernel. Developing a specialized string kernel for AAC features is a direction of future research.

The value of AAC is evident for protein pairs in which one or both proteins have no domain information. Using a classifier with amino acid composition we were able to predict interactions of several uncharacterized proteins and were able to predict novel function for some of these proteins based on the known annotation of their interacting partners.

The post-hoc analysis of why a simple feature like AAC works so well by itself showed that several of the AAC features were significantly over-represented in domains involved in protein-protein interactions. This indicated that AAC features are likely capturing crucial contact points of the protein domains, and therefore helping in prediction. Although amino acids have been previously shown to have differential concentration in different interaction surfaces [28], [29], our work extends this analysis to assessing importance of amino acids in a dataset-specific manner. We found that the importance of amino acids depended on the particular dataset, which maybe due to the propensity of different assays to capture different classes of interactions (e.g. transient versus stable).

We found that AAC features constitute a non-trivial fraction (26–40%) of the 100 most important features in a classifier using both AAC and domains as features. If protein domains were capturing all the properties of interacting proteins, we would not expect AAC features to be important when used with domains. This is further supported by the observation that only a subset of the AAC features important for interaction prediction were enriched in known interacting domains. This suggests the possibility of certain properties of interacting surfaces that are not fully captured in algorithms that search only for protein domains. AAC features can provide a cue for detecting novel types of protein domains encoding meta-level information important, possibly for docking of a protein partner or presentation of the interaction domain. Such meta-interaction surfaces identified with high confidence can be experimentally verified, leading to the identification of new types of protein domains, that may not be necessarily linear.

Our prediction results using simple amino acid composition have been quite encouraging, and has opened a plethora of questions regarding the information that can be captured at the level of single and pairs of amino acids. Extending this work to recognize higher-order signals in the proteome, including identification of meta-domains, can provide insight into the causal and mechanistic details of protein interactions.

## Methods

### Feature extraction

Prior to prediction of protein interactions, we represent every protein pair in our datasets using binary feature sets corresponding to attributes of protein pairs. These features correspond to AAC features and domain features.

### AAC as classifier features

We use two types of features for representing AAC: monomer and dimer features. Monomer features capture composition of individual amino acids, whereas dimer features capture composition of pairs of consecutive amino acids. To generate the monomer features, we first obtain a 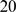-dimensional vector, 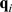, representing the proportion of the 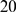 amino acids in a protein, 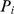. Each dimension, 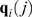, is the count of a particular amino acid in 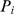, normalized by the length of 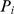. The real-valued composition is discretized into 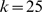 bins, producing a set of 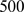 binary features, with 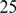 features per amino acid. The number of bins for discretization is determined on a hold-out set (Supporting text S1, Fig. S1, S2).

To generate the dimer features, we obtain a 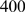-dimensional vector of all possible pairs of amino acids that can be extracted from the protein sequence. Similar to the monomer composition we normalize the dimer composition by 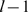, where 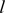 is the length of the protein, followed by discretization into 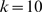 bins, which is determined empirically on a hold-out set (Supporting text S1, Fig. S3).

### Domains as classifier features

The domains are represented as binary features, with each feature identified by the domain name. For yeast proteins, we use domains that are available for download from the Saccharomyces genome database. For fly and worm proteins, we used interproscan domains [30].

### Description of other non-domain features

We have compared the AAC features against other non-domain, sequence-based features. These features are the tuple features [10] and signature products [14]. The tuple features were created by first grouping amino acids into six categories based on their bio-chemical properties, and then creating all possible strings of length 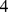 using these six categories.

The signature products are used directly within a support vector machine (SVM) framework where protein pairs are represented using a specialized signature product kernel. This approach first extracts signatures of length 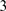 from the individual protein sequences. Each signature consists of a middle letter and two flanking amino acids represented in alphabetical order. Thus two 3-tuples with the first and third amino acid letter permuted have the same signature. For example the 3-tuples ATC and CTA are both represented by the signature T(AC). The signatures are used to construct a *signature kernel* specifying the inner product between two proteins. The signature kernel itself is used in a tensor product to define the signature product kernel between pairs of proteins.

### Protein interaction prediction via binary classification

Prediction of protein interactions via binary classifiers is a well-known approach, briefly outlined here. In this approach, each data point corresponds to a protein pair 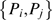, which is associated with a binary class random variable, 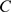, taking two values, 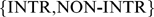. Each protein pair is represented as binary feature vector 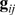, which is obtained from the OR of individual feature vectors, 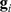 and 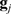, of the two proteins 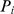, respectively: 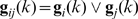, where 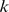 is an index over the feature set. This allows protein pairs 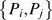 and 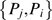 to be treated symmetrically, that is 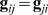, 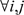.

Protein pairs that represent interacting pairs have 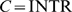, and protein pairs representing non-interacting pairs have 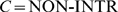. We use three types of classifiers well known in the machine learning literature: maximum entropy classifier (also known as logistic regression classifier), support vector machines, and the Naive Bayes classier.

### Maximum entropy classifier

A maximum entropy classifier is a probabilistic classifier for binary classification [31], estimating two conditional probability values, 
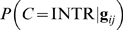
 and 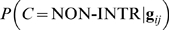 for each protein pair 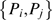, where 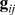 is the binary feature vector associated with 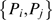. The first conditional probability value describes the probability of the proteins, 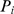 and 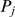, to interact, and the second conditional probability describes the probability of these proteins to not interact. Specifically, the conditional probability of interacting is:
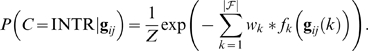



Here, 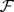 is the set of 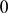-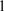-valued feature functions, 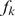, 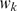 is the weight of 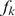, 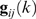 is the value of the 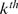 feature in 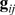, and 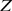 is a normalization term. The feature functions, 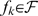 correspond to attributes of protein pairs, and return 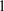 if the attribute is true, and 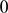 if the attribute is false. 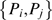 is classified into INTR if 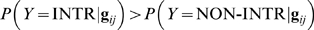. We used the maximum entropy classifier from the Mallet toolkit [32].

### Naive Bayes classifier

A naive Bayes classifier is similar to a maximum entropy classifer in that it uses the class conditional distribution to assign a protein pair to the interacting (INTR) or non-interacting (NON-INTR) class. However, the form of conditional distribution is given by
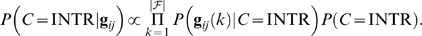



The proportionality term, 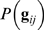 is the same for 
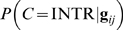
 and 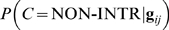 and is not required for class prediction. We used the Weka toolkit for the Naive Bayes classifier [33].

### Support vector machine

A support vector machine (SVM) classifier does not estimate class conditional distributions, but rather a maximum margin hyper-plane between the positive and negative examples of the class. The hyper-plane is defined by a set of *support vectors*, and a data point is classified as INTR or NON-INTR using a set of inner products with the support vectors. Most real-world data are not linearly separable in the input feature space requiring non-linear classifiers that project the input data into a high-dimensional space where the data is separable. In SVMs, because all computations on the input data are written as inner products, a kernel function is typically used to efficiently compute inner products in a high-dimensional space. We used the SVM classifier with a radial basis kernel (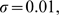 Supporting text S1, Fig. S1) from the SVMLight toolkit [34].

### Datasets

We analyzed several protein interaction datasets from yeast, worm and fly (Table 4). We used three datasets for yeast, *S. cerevisiae*, each capturing different types of interactions based on the experimental assay: TWOHYB (Yeast Two-hybrid), AFFMS (Affinity pull down with mass spectrometry) and PCA (protein complementation assay). TWOHYB and PCA comprise pairwise interactions, whereas AFFMS comprises co-complex interactions. We treated these three datasets separately to avoid confounding factors across different experimental assays. These were obtained from the General Repository for Interaction Datasets (GRID) database [35]. We had one dataset each for worm, *C. elegans* [4] and fly, *D. melanogaster* [35], referred to as WORM and FLY respectively. Both worm and fly datasets were generated from Two-hybrid assays.

**Table 4:** Description of datasets.

Dataset	Interactions with domains	Interactions without domains
AFFMS	22183	2409
TWOHYB	6038	1156
PCA	2288	292
WORM	14233	972
FLY	66259	4052

All datasets other than WORM were obtained from [35]. WORM was obtained from [4].

We obtained the amino acid sequence for yeast proteins from the Saccharomyces genome database (SGD, http://www.yeastgenome.org), worm proteins from Wormbase (version WormPep168, http://www.wormbase.org) and fly proteins from the Integr8 portal at the European Bioinformatics Institute (EBI, http://www.ebi.ac.uk/integr8/FtpSearch.do?orgProteomeId=17). Interproscan domains for worm proteins were obtained via the Wormmart program (http://www.wormbase.org/biomart/martview). Fly protein domains were obtained from EBI, and yeast domains were downloaded from SGD, which in turn stores domain match results of yeast proteins using Interproscan [36].

We pre-processed each dataset to ensure that the number of positive and negative examples in a dataset were equal. We retained self-interacting pairs in the positive set to allow the prediction of such interactions in the test set. We empirically verified that the self-interacting pairs did not influence classifier performance (Fig. S5). Negative datasets were generated using the *closed world assumption* that all protein pairs that were not in the positive set were in the negative set. We then generated a negative set of size equal to the positive set by drawing protein pairs from the complement of the positive set. Although negative sets have been created based on the co-localization of proteins, uniform sampling of non-interacting protein pairs has been shown to produce unbiased estimates of the true distribution of negative examples [37].

### Classifier performance evaluation

We evaluated the classifiers using the receiver-operator characteristic (ROC) curves that compared the sensitivity of the classifier as a function of the false positive rate [38]. Sensitivity is the ratio of the number of correctly predicted interacting pairs (true positive, TP), to the size of the complete interacting set (positive, P). False positive rate is the ratio of the number of incorrectly predicted interacting pairs (false positive, FP), to the size of the complete non-interacting set (negative, F). The area under the ROC curve (AUC), *AUC-score*, estimates the overall classifier performance. We performed five-fold cross-validation for the datasets, and report the mean and standard deviation of the AUC-score computed on the test set of each fold of validation. We use 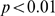 as a threshold for statistical significance for comparing classifiers with different features.

### Analysis of important AAC features

To assess which features were most beneficial for our predictions, we computed a feature importance score, 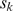 for each feature 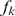 as follows
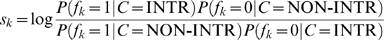
(1)


This score assigns a high positive value to a feature 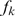, if it is present (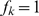) among protein pairs labeled as INTR and is absent among protein pairs labeled as NON-INTR. We identified the set of important features from the three yeast datasets, AFFMS, TWOHYB and PCA, independently. For each dataset we considered the true positives in the top 500, 1000, 1500 or 2000 predicted interactions, and computed the importance for all the features that occurred in these true positives. We then selected the top 100, 200 and 300 features using the feature importance score to identify the most important features.

### Enrichment of AAC features in domains known to be involved in protein interactions

We obtained the Interproscan IDs of domains predicted to be involved in protein interactions with high confidence from the Domine database (http://domine.utdallas.edu/cgi-bin/Domine [20]). We then obtained from SGD the start and stop positions of these domains in yeast proteins. To assess enrichment of a given AAC dimer 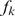 in the domain regions, we used the Hypergeometric distribution with parameters, 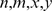:
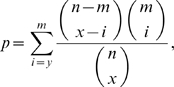
where 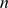 is the total number of occurrences of all dimers in the proteins, 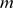 is the total number of occurrences of 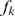 in the proteins, 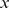 is the total number of occurrences of all dimers in the domain regions, and 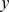 is the total number of occurrences of 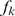 in the domain regions. The 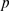-value tells us how likely it is to see 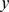 or more occurrences of dimer 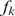 in 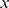 random draws from a pool of 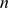 dimers, of which 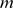 are of type 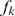. A similar technique was used to assess enrichment of AAC monomer features. We use 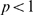
E-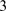 as a threshold for significance.

## Supporting Information

Text S1 Supplementary file describing methods the discretization of Amino acids, other methods of enrichment analysis, additional results of the MAXENT classifier, and performance of classifiers with and without self-interacting proteins in the positive set.
(0.05 MB PDF)

Figure S1 SVM AUC means as a function of increasing number of bins (k) for obtaining the AAC monomer features. The standard deviations varied in the range [0.002–0.07]
(0.33 MB TIF)

Figure S2 AUC means of the three classifiers (SVM, Maximum Entropy, NaiveBayes) as a function of increasing number of bins (k). The standard deviations varied in the range [0.02–0.06] for SVM, [0.02–0.07] for Naive Bayes, and [0.02–0.05] for Maximum entropy classifiers.
(0.30 MB TIF)

Figure S3 AUC mean of SVM classifier as a function of increasing number of bins (k) for obtaining the AAC dimer features.
(0.24 MB TIF)

Figure S4 Maximum Entropy classifier performance using AAC or tuple features on protein pairs with and without domains, and the complete dataset.
(0.69 MB TIF)

Figure S5 Performance comparison of the SVM classifier with or without the self-interacting proteins. Classifiers used either AAC monomer or domains as features.
(0.43 MB TIF)
