# Supplementary material for: Exploiting Amino Acid Composition for Predicting Protein-Protein Interactions
Source: PLoS One. 2009 Nov 20;4(11):e7813. doi: 10.1371/journal.pone.0007813 (PMC2775920; doi:10.1371/journal.pone.0007813)
Supplement: Text S1 — Supplementary file describing methods the discretization of Amino acids, other methods of enrichment analysis, additional results of the MAXENT classifier, and performance of classifiers with and without self-interacting proteins in the positive set. (0.05 MB PDF) [file pone.0007813.s001.pdf]

Supplementary material

1 Selection of number of bins k

We tuned k on a hold-out set of 4000 protein pairs generated by selecting protein pairs uniformly at random from the complete GRID dataset. The GRID dataset includes interacting protein pairs from PCA, Two-hybrid and Affinity-MS assays. These protein pairs were not included for any of the AUC numbers reported in the manuscript. Using the support vector machine (SVM) as a classifier we obtained mean area under the curve (mean AUC) scores as a function of k, $5 \leq k \leq 45$, where k was used as the number of bins for generating the AAC monomer features (Fig S1). We found that at $k = 20$ and $k = 25$, the SVM classifer performance was the best. We also used this hold-out set for selecting σ, which was varied between 0.005 and 0.5. We found the performance of the SVM to be similar for $\sigma \in \{0.005, 0.01, 0.05\}$, and selected $\sigma = 0.01$ for all the experiments in the manuscript. $k = 25$ for the number of bins was also close to optimal for the Maximum Entropy and the Naive Bayes classifiers (Fig S2).

The number of bins for dimer features was also obtained using the SVM classifier on the hold-out set (Fig S3). The mean AUC peaked at $k = 10$ and this was used as the number of bins for the dimer features.

2 Statistical significance analysis for co-location, co-process and co-function

To assess the statistical enrichment of co-location of our predicted interactions, we used the hyper-geometric distribution to give the probability of observing k_c or more, out of K_c predicted interactions, to have co-location, given there are l_c out of L protein pairs that are co-located. Here k_c is the number of predicted interactions at confidence c, $c \in \{0.95, 0.90, 0.85, 0.80\}$, that also have co-location; K_c is the number of predicted interactions at confidence c for which both proteins have gene-ontology annotation; L is the number of possible protein pairs that have associated gene-ontology information; l_c is the number of protein pairs from the set of L that exhibit co-location at confidence c. We estimate the values of k_c, l_c, and K_c for each confidence level, c for both predicted interactions and non-interactions. Statistical depletion was estimated by the probability of observing $\leq k_c$ predicted interactions with co-location out K_c total predicted interactions under the hyper-geometric distribution. A similar procedure was used for co-process and co-function for both interactions and non-interactions.

3 Comparison of AAC against non-domain features using Maximum entropy

We compared AAC against other non-domain features such as tuples and signature product (Fig S4). Results on the SVM classifier are reported in the manuscript. Here we describe the results on a maximum entropy classifier. On protein pairs for which no domain information was available we found AAC monomer and dimer features to significantly outperform the tuple feature on the AFFMS dataset. Although tuple features

had a higher performance than AAC monomer on the PCA dataset, this was not statistically significant. Surprisingly, AAC monomer also significantly outperformed AAC dimer on the AFFMS dataset for protein pairs with no domains. This indicates to the importance of the overall R-group characteristics in protein interactions.

4 Comparison of protein interaction prediction results with and without self interactions

To determine if self-interacting proteins were affecting the performance of the classifiers using AAC-based features, we compared the performance of the SVM classifier on datasets with the self-interacting proteins removed (Fig S5). We compared classifiers using AAC monomers and domains and found that the presence of the self-interacting proteins does not affect performance. Specifically, the AUC scores of classifiers with or without the self-interacting proteins is statistically indistinguishable.
