# Supplementary figures and images for: Exploiting Amino Acid Composition for Predicting Protein-Protein Interactions
Source: PLoS One. 2009 Nov 20;4(11):e7813. doi: 10.1371/journal.pone.0007813 (PMC2775920; doi:10.1371/journal.pone.0007813)

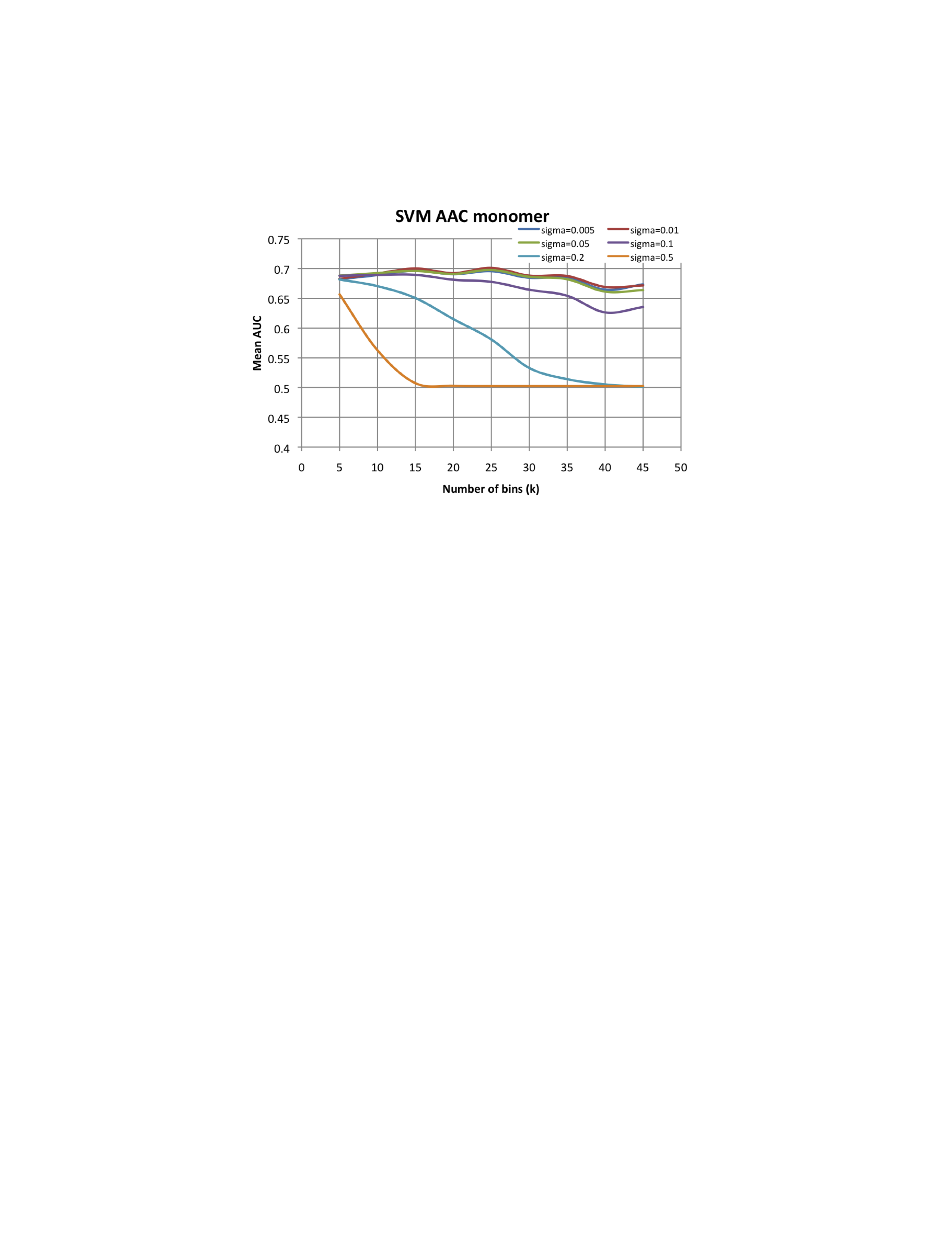

Supplement: Figure S1 — SVM AUC means as a function of increasing number of bins (k) for obtaining the AAC monomer features. The standard deviations varied in the range [0.002–0.07] (0.33 MB TIF) [file pone.0007813.s002.tif]

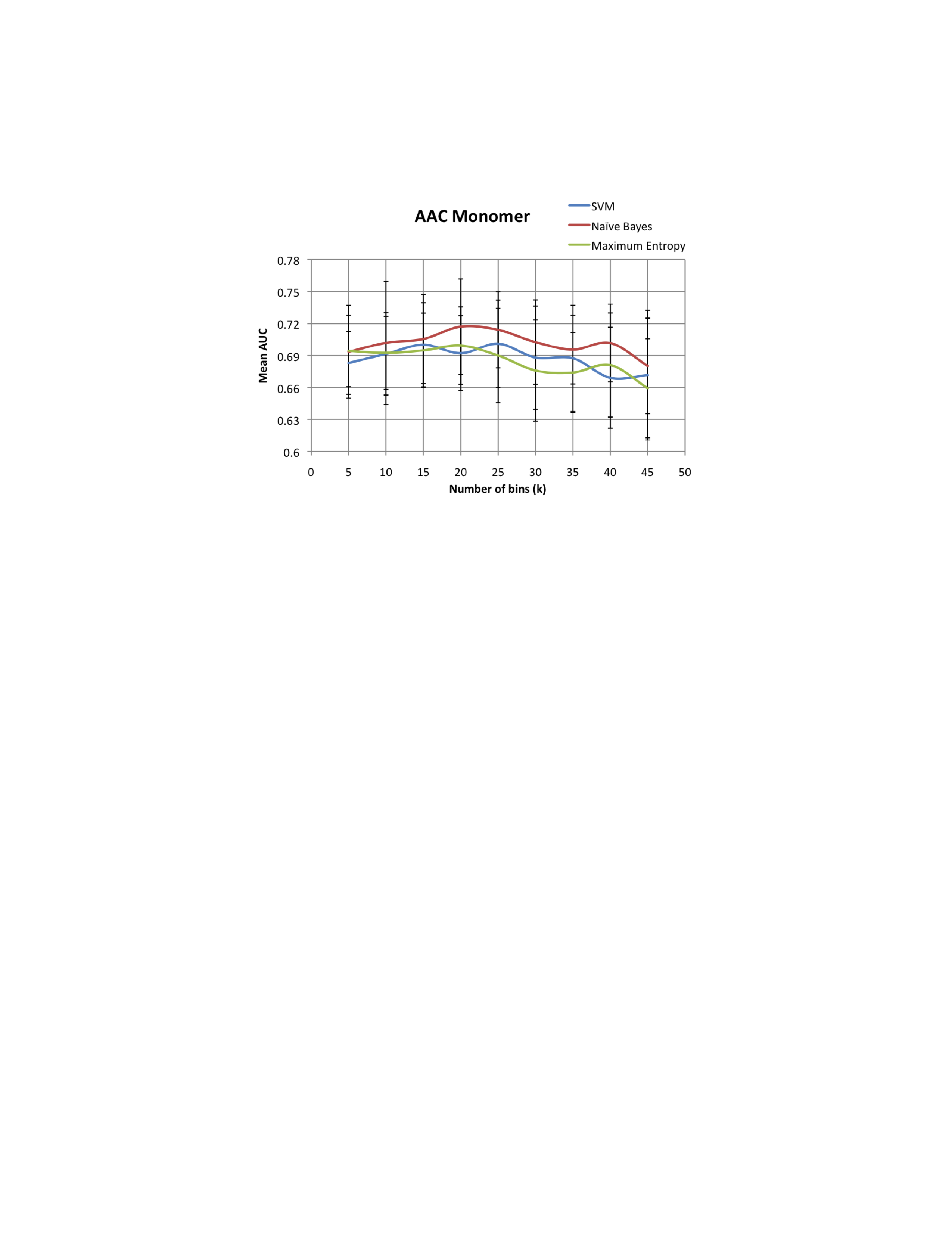

Supplement: Figure S2 — AUC means of the three classifiers (SVM, Maximum Entropy, NaiveBayes) as a function of increasing number of bins (k). The standard deviations varied in the range [0.02–0.06] for SVM, [0.02–0.07] for Naive Bayes, and [0.02–0.05] for Maximum entropy classifiers. (0.30 MB TIF) [file pone.0007813.s003.tif]

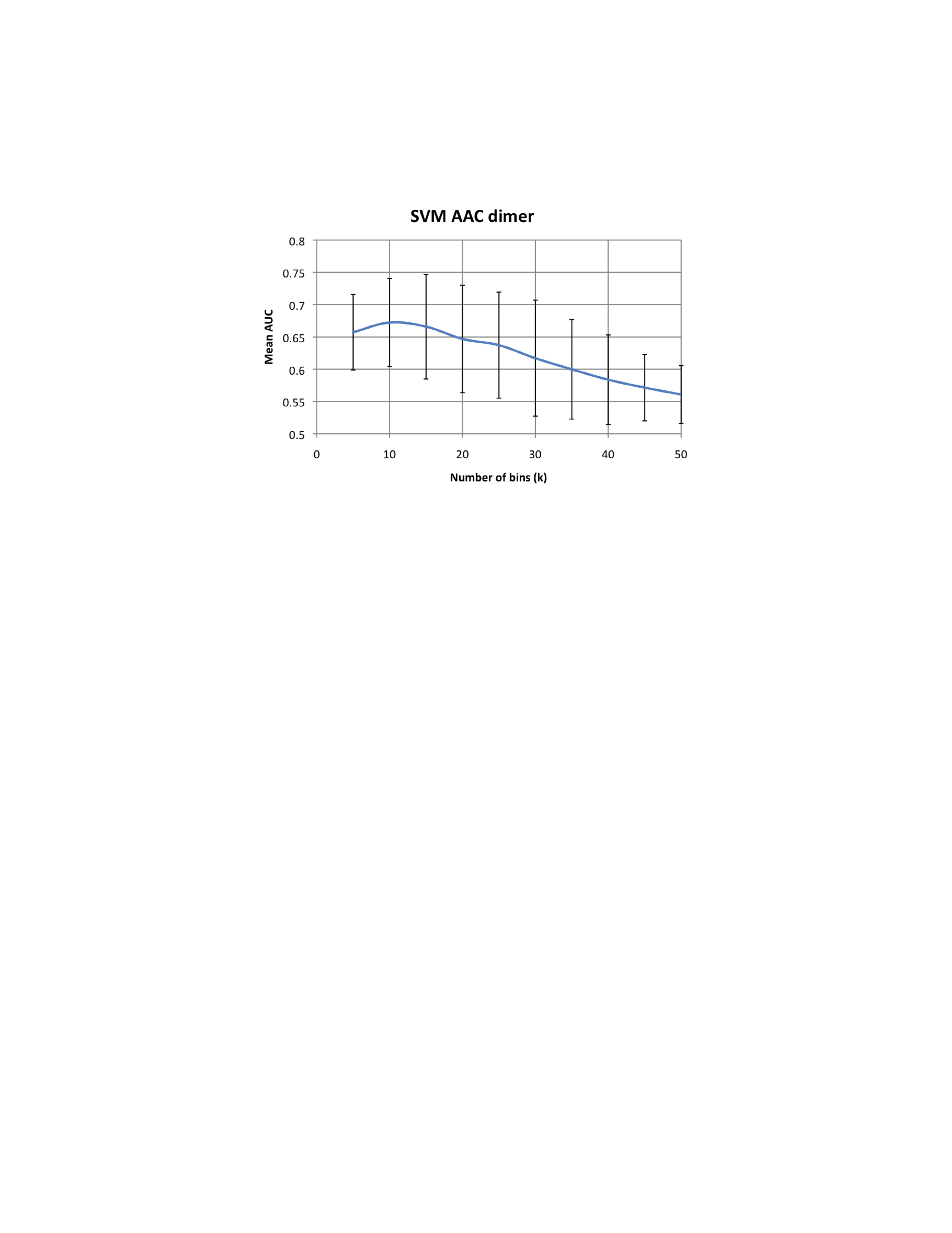

Supplement: Figure S3 — AUC mean of SVM classifier as a function of increasing number of bins (k) for obtaining the AAC dimer features. (0.24 MB TIF) [file pone.0007813.s004.tif]

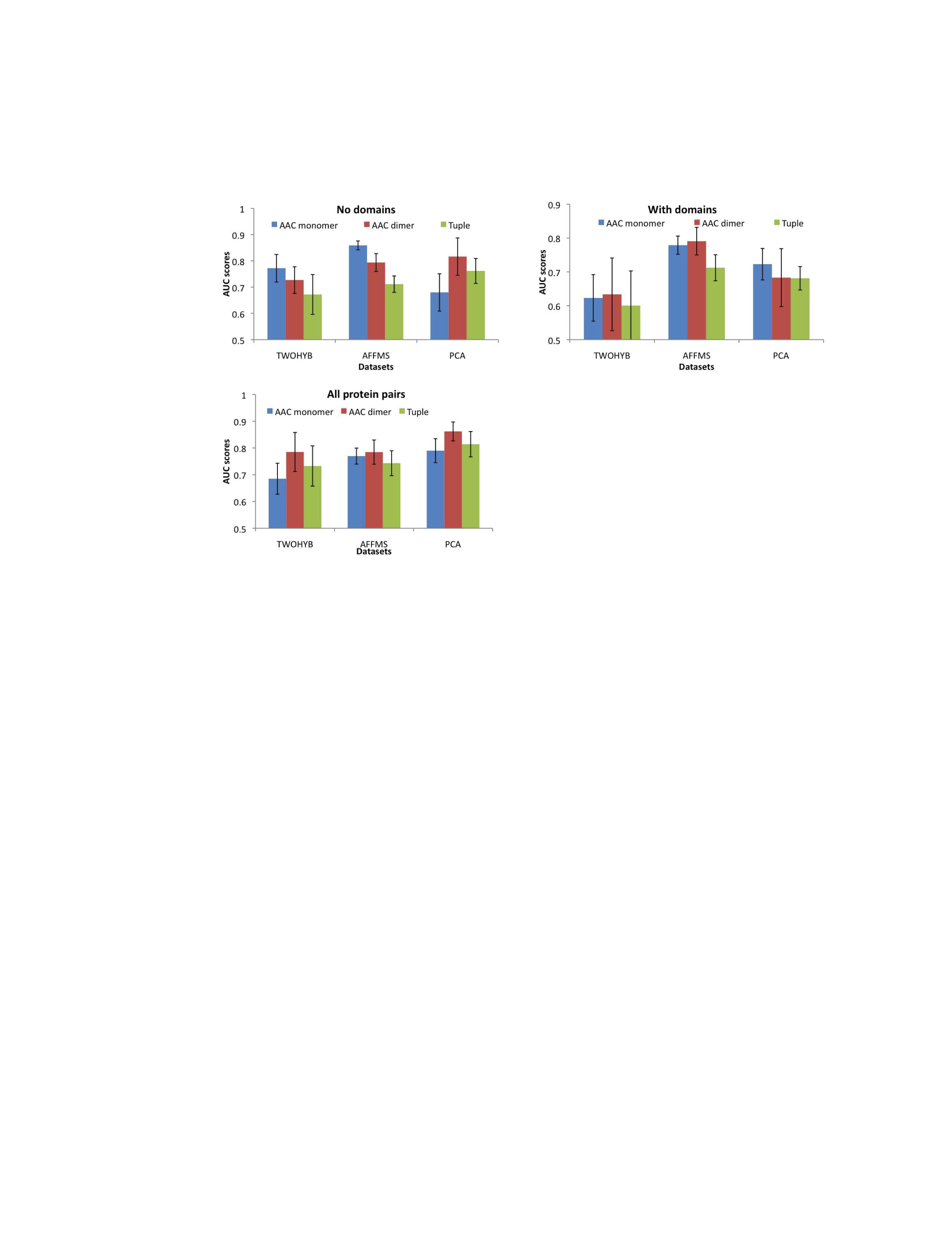

Supplement: Figure S4 — Maximum Entropy classifier performance using AAC or tuple features on protein pairs with and without domains, and the complete dataset. (0.69 MB TIF) [file pone.0007813.s005.tif]

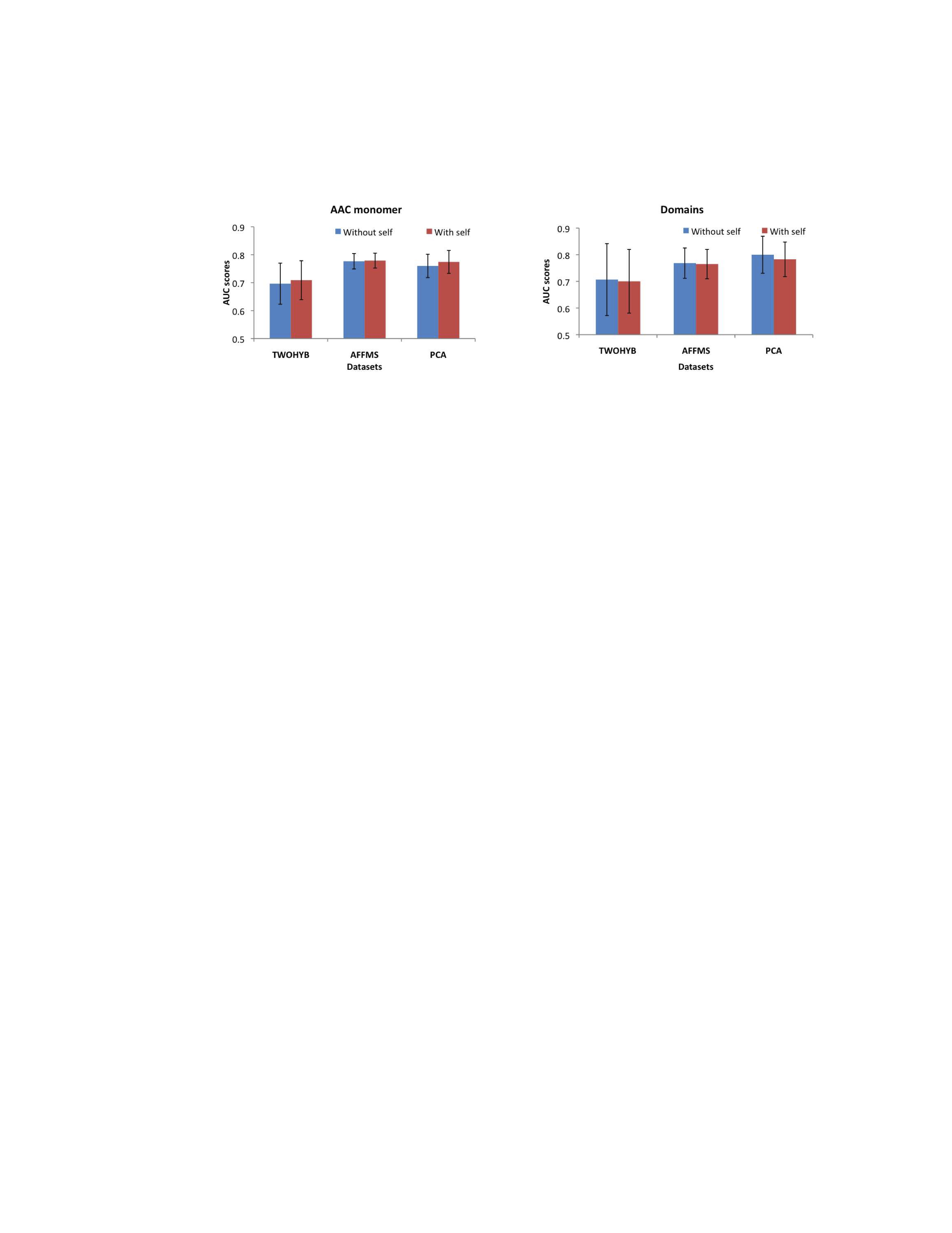

Supplement: Figure S5 — Performance comparison of the SVM classifier with or without the self-interacting proteins. Classifiers used either AAC monomer or domains as features. (0.43 MB TIF) [file pone.0007813.s006.tif]
